# Parenting stress among parents of children with congenital diaphragmatic hernia

**DOI:** 10.1007/s00383-017-4093-4

**Published:** 2017-05-19

**Authors:** Elin Öst, Margret Nisell, Björn Frenckner, Carmen Mesas Burgos, Maria Öjmyr-Joelsson

**Affiliations:** 1 Karolinska Institutet, Department of Women’s and Children’s Health, 171 76 Stockholm, Sweden; 2 Pediatric Surgery Unit, Karolinska University Hospital, Astrid Lindgren Children’s Hospital, 171 76 Stockholm, Sweden; 3 The Red Cross University College, Huddinge, Sweden

**Keywords:** Parental stress, Congenital diaphragmatic hernia, Long-term follow-up, Extracorporeal membrane oxygenation, Prenatal diagnosis

## Abstract

**Purpose:**

The aim of this study was to examine parental stress among parents of children with congenital diaphragmatic hernia (CDH).

**Methods:**

Between 2005 and 2009, a total of 51 children with CDH were treated at Astrid Lindgren Children’s Hospital. The survival rate at discharge was 86% and long-term survival rate 80%. One parent each of the long-term survivors (41 children) was included in the present study, and 34 parents (83%) agreed to participate. Participants received the Swedish Parenthood Stress Questionnaire (SPSQ). The questionnaire was supplemented by data from case records.

**Results:**

Parents of children with CDH, who had been supported by ECMO or had a long hospital stay, showed significantly higher overall parental stress. Mothers scored an overall higher parental stress compared with fathers. A prenatal diagnosis of CDH or lower parental educational level resulted in significantly higher parental stress in some of the factors.

**Conclusions:**

Parental stress in parents of children with CDH seems to increase with the severity of the child’s malformation. Mothers tend to score higher parental stress than fathers.

## Background

Congenital diaphragmatic hernia (CDH) is a life-threatening anomaly which occurs in 2–3:10,000 pregnancies [1]. Due to a defect in the diaphragm, abdominal viscera herniate into the thoracic cavity and babies are born with small and hypoplastic lungs [2]. Early management strategies, such as preoperative stabilization, gentle ventilation and access to extracorporeal membrane oxygenation (ECMO), have lead to an increased survival rate [3–7]; in our institution, 85% [8]. However, in follow-up studies, centers report long-term sequelae such as failure to thrive, gastrointestinal disease, pulmonary morbidity, cardiovascular disease, hearing loss, neurocognitive defects, chest asymmetry, and scoliosis [9–16].

The vast majority of pregnant women in the Western world undergo ultrasound examination of the fetus. In Sweden, more than 95% are routinely examined [17]. The prevalence of birth defects among Swedish children is 3%, and approximately 0.5% of all pregnancies lead to a termination due to the diagnosis of a fetal anomaly [1]. A detection of a fetal anomaly often leads to acute psychological parental distress, where the severity of the anomaly, gestational age, and diagnostic and prognostic ambiguity are strong predictors of severe psychological distress [18]. A healthy transition to parenthood is influenced by fetal and future child health, and how information about a fetal anomaly during counseling is presented has a great importance in the relationship between the parent and the baby [19].

The number of prenatally detected fetuses with CDH has increased over the last few years, giving rise to new questions and alternatives for both caregivers and parents. A prenatal diagnosis often indicates a more severe case of the malformation with a higher mortality risk [20]. Even though a prenatal diagnosis of CDH allows for consultations with specialists and planning for optimal delivery, it may lead to increased psychological distress for the parents [21].

Expecting a child and the transition into parenthood have a strong impact upon most parents. In normal pregnancies, a woman’s physical and emotional self-rated health has been described as being negatively affected by pregnancy and the first year of motherhood. For new fathers, however, their health is stable throughout the pregnancy and the postpartum period, but negatively affected by the first year of parenthood [22]. Parental stress can be defined as “an adverse psychological reaction to the demands of being a parent” [23] or “a notion of conflict between parental resources and the demands connected to the parental role” [24].

There are various factors that contribute to the level of parental stress such as general health, anxiety and psychological problems [25]. Social background, employment, educational level and being a mother have also been described as strongly associated contributing factors for a higher level of parental stress [26].

The aim of this study was to examine parental stress among parents of children with CDH.

## Methods

### The parents and their children

Between the years 2005 and 2009, a total of 51 children with CDH were treated at Astrid Lindgren Children’s Hospital in Stockholm. The survival rate at discharge was 86, and 80% (41 children) were, according to the Swedish population register, alive at the time of the follow-up (long-term survival rate). One parent of each of the long-term survivors, mother or father, was invited by mail to participate in the present study. Data on gender, prenatal diagnosis, birth weight, gestational age, side of lesion, method of surgical repair, age at surgery, time to intubation, history of ECMO treatment and type of discharge from hospital were collected from the case records. The parent’s educational degrees were obtained from another questionnaire that was sent to the families at the same time.

### Questionnaire

The Swedish Parenthood Stress Questionnaire (SPSQ) was used to measure the stress that parents can relate to parenting and was administrated by mail as a hard copy. The Swedish Parenthood Stress Questionnaire is based on the American questionnaire Parenting Stress Index (PSI) by Abidin [27] and was translated and adjusted to a Swedish population by Östberg et al. [28]. They concluded that the SPSQ could be used as a reliable and valid instrument for measuring experienced parental stress [28]. The Swedish version contains 34 questions within five factors: Incompetence regarding Parenthood (general experiences of caregiving, feelings of incompetence in the parental role and the difficulties of parenthood), Restrictions of Role (parental responsibilities), Social Isolation (social contacts outside the family), Relationship with Spouse (social experiences within the family) and Parental Health (physical fitness, infections and fatigue). Answers are made through a five-point Likert-type response scale on which the parents mark to what extent they agree or disagree with the statement presented. High values indicate a high level of stress.

A total score for all the study participants was calculated, and for identifying any potential risk factors for parental stress within the group, data were divided into subgroups and compared thus: parents with/without a prenatal diagnosis of CDH, parents of children treated with/without ECMO, parents with a higher/lower education, parents of children with a long/short hospital stay (divided by the median), parents of children with CDH born 2005–2006/2007–2009 (younger vs. older children at the time of filling out the questionnaire) and according to who had answered the questionnaire (mothers/fathers).

A Swedish nationwide representative sample, which has earlier been described by Östberg et al., when developing the questionnaire, was used as a reference [28].

### Ethics

This study was approved by the regional ethical committee in Stockholm, Dnr 2011/472-31/4. Written informed consent was obtained from all individual participants included in the study.

### Statistics

Data are presented as mean ± SD, median, absolute values (*n*) and frequencies (%) for categorical variables. For numerical data, the Mann–Whitney *U* test for independent samples was used for comparing differences regarding parental stress between subgroups. Fisher’s exact test was used to compare differences between the groups. Significance was set at *p* < 0.05; however, the exact *p* values are presented in tables for transparent reader assessment of associations potentially arising by chance.

## Results

### Participants and patient characteristics

A total of 34 parents (83%) agreed to participate in the study, while 7 declined actively or passively. Out of the 34 who were willing to participate, 6 never completed the questionnaire and 1 returned the questionnaire incomplete and was thus excluded. The final number of participants was 27 parents (66%): 21 mothers and 6 fathers. There were no significant differences among the children of the study participants and non-participants, who had declined to participate or had been excluded from the study, regarding gender, prenatal diagnosis, birth weight, gestational age, side of lesion, method of surgical repair, age at surgery, time to intubation, history of ECMO support or type of discharge from hospital (Table 1). However, among the non-participants, there were differences between those whose children were deceased and those who declined to participate. The deceased children had undergone a patch repair, were intubated immediately after birth and needed ECMO support more frequently than the children of the study participants and the other non-participants. Eight families in the group of non-participants, compared with six in the study group, had been referred to another hospital and did not attend our long-term follow-up program. For further characteristics, see Table 1.

**Table 1:** Demographic data for all children with CDH treated at Astrid Lindgren Children’s Hospital 2005–2009 (both children of study participants and non-participants) *n* (%)

	Entire cohort *n* = 51	Study participants *n* = 27	Non-participants *n* = 24	
			Declined or excluded *n* = 14	Deceased *n* = 10
Gender				
Male	33 (65)	17 (63)	10 (71)	6 (60)
Female	18 (35)	10 (37)	4 (29)	4 (40)
Prenatal diagnosis	28 (55)	15 (56)	6 (43)	7 (70)
Birth weight (kg) (mean ± SD)	3.3 ± 0.7	3.5 ± 0.7	3.1 ± 0.6	3.3 ± 0.8
Gestational age (weeks) (mean ± SD)	38 ± 2	38 ± 2	38 ± 3	38 ± 1
Side of lesion				
Left	45 (88)	24 (89)	13 (93)	8 (89)
Right	5 (10)	3 (11)	1 (7)	1 (11)
Bilateral	0 (0)	0 (0)	0 (0)	
Repaired				
Primary	19 (37)	11 (31)	8 (57)	0 (0)*
Patch	31 (61)	16 (59)	6 (43)	9 (90)*
No repair	1 (2)	0 (0)	0 (0)	1 (10)
Age at surgery (h) (median)	96	96	120	120
Intubated within 6 h from birth	39 (76)	21 (78)	8 (57)	10 (100)
ECMO	22 (43)	12 (44)	3 (21)	7 (70)
ECMO > once	7 (14)	3 (11)	2 (14)	2 (20)
LOS (days) (mean, min–max)		55 (5–304)	28 (5–84)	
Referred to other hospital		6 (22)	8 (57)	
Survival to discharge	44 (86)	27 (100)	14 (100)	3 (30)*
Long-term survivors (2012)	41 (80)	27 (100)	14 (100)	0 (0)*

* *p* < 0.05, when compared with study participants

### Results from the Swedish Parenthood Stress Questionnaire

The total score of all five subscales in the questionnaire for all parents resulted in *M* = 2.26 (SD 0.58); Incompetence regarding Parenthood *M* = 1.91 (SD 0.63), Restrictions of Role *M* = 3.16 (SD 0.81), Social Isolation *M* = 1.92 (SD 0.78), Relationship with Spouse *M* = 2.12 (SD 0.95) and Parental Health *M* = 2.44 (SD 0.84) (Table 2). Parents whose children had required ECMO support reported a total mean of 2.51 and a mean of 3.49 regarding Restrictions of Role. Parents of children with CDH who required versus not required ECMO treatment had a significantly higher level (*p* = 0.03) of parental stress in general, and within the Parental Health factor in particular (*p* = 0.05), when compared with the former group (Tables 3, 4).

**Table 2:** Parental stress among all participating parents of children treated for CDH 2005–2009

Variable	Median	*N*	Mean	SD	Minimum	Maximum
Total SPS Qscore	2.24	27	2.26	0.58	1.35	3.97
Incompetence	1.64	27	1.91	0.63	1.09	3.55
Role Restriction	3.14	27	3.16	0.81	1.43	4.86
Social Isolation	1.71	27	1.92	0.78	1.00	4.43
Spouse Relationship	1.60	27	2.12	0.95	1.00	4.80
Health Problems	2.25	27	2.44	0.84	1.50	4.50

Age of child: *m* = 55 months, SD = 18 months, range 25–95 months

**Table 3:** Comparison of responses to parental stress among parents of children born with CDH

Comparative groups	Treated with vs. without ECMO		Prenatal vs. postnatal diagnosis		Higher vs. lower education		Mothers vs. fathers		Long vs. short LOS		2005–2007 vs. 2007–2009	
	*n* = 12/*n* = 15	*p* value	*n* = 15/*n* = 12	*p* value	*n* = 13/*n* = 14	*p* value	*n* = 21/*n* = 6	*p* value	*n* = 13/*n* = 14	*p* value	*n* = 12/*n* = 15	*p* value
Total SPSQ score	2.51/2.06	0.03*	2.32/2.19	0.5	2.05/2.46	0.07	2.37/1.89	0.04*	2.49/2.05	0.04*	2.27/2.26	0.5
Incompetence	2.08/1.76	0.1	1.87/1.95	0.2	1.71/2.08	0.06	2.04/1.44	0.005*	2.08/1.75	0.08	1.98/1.84	0.1
Role Restriction	3.49/2.90	0.07	3.35/2.93	0.1	3.14/3.18	0.4	3.37/2.43	0.007*	3.46/2.89	0.07	3.04/3.27	0.1
Social Isolation	3.18/1.71	0.06	2.00/1.82	0.3	1.63/2.19	0.03*	1.93/1.88	0.4	2.11/1.74	0.1	1.90/1.93	0.2
Spouse Relationship	2.40/1.89	0.08	2.01/2.25	0.3	1.66/2.54	0.01*	2.12/2.10	0.2	2.35/1.90	0.06	2.27/2.00	0.2
Health Problems	2.71/2.23	0.05*	2.72/2.10	0.03*	2.27/2.61	0.2	2.58/1.96	0.07	2.75/2.16	0.03*	2.35/2.24	0.3

Listed *p* values are based on comparisons of the groups using the Mann–Whitney test; *p* < 0.05 is considered significant

**Table 4:** Comparison of SPSQ total scores among parents of children born with CDH

	SPSQ total scores						
							Mann–Whitney
	*n*	Mean	SD	Min	Max	Median	*p* value
Prenatal diagnosis							
Yes	15	2.32	0.66	1.35	3.97	2.18	0.5
No	12	2.19	0.46	1.35	2.65	2.47	
ECMO							
ECMO	12	2.51	0.63	1.53	3.97	2.53	0.03*
No ECMO	15	2.06	0.46	1.35	2.88	2.18	
Parent							
Mother	21	2.37	0.57	1.44	3.97	2.27	0.04*
Father	6	1.89	0.47	1.35	2.53	1.74	
Length of hospital stay							
Short	14	2.05	0.47	1.35	2.88	2.09	0.04*
Long	13	2.49	0.61	1.53	3.97	2.53	
Educational level							
High	13	2.05	0.46	1.35	2.88	2.00	0.07
Low	14	2.46	0.62	1.53	3.97	2.49	
Childs age							
Older	12	2.27	0.67	1.35	3.97	2.35	0.5
Younger	15	2.26	0.50	1.44	3.32	2.24	

Listed *p* values are based on comparisons of the groups using Mann–Whitney test; *p* < 0.05 is considered significant

Being a mother of a child with CDH was a single significant predictor of a higher level of total parental stress (*p* = 0.04). The distinguishing factors were Incompetence regarding Parenthood (*p* = 0.005) and Restrictions of Role (*p* = 0.007), where mothers had a strongly significant higher parental stress compared with fathers (Tables 3, 4). There were no significant differences in background parameters between mothers and fathers (Table 5).

**Table 5:** Background characteristics for mothers and fathers

	Parent						
	Father		Mother		Total		Fisher’s test
	*n*	Col (%)	*n*	Col (%)	*n*	Col (%)	*p* value
Educational level							
High	2	33.3	11	52.4	13	48.1	0.648
Low	4	66.7	10	47.6	14	51.9	
ECMO							
ECMO	3	50.0	9	42.9	12	44.4	1.000
No ECMO	3	50.0	12	57.1	15	55.6	
Repair							
Patch	4	66.7	12	57.1	16	59.3	1.000
Primary	2	33.3	9	42.9	11	40.7	
Length of hospital stay							
Short	3	50.0	11	52.4	14	51.9	1.000
Long	3	50.0	10	47.6	13	48.1	
Discharge							
Another hospital	1	16.7	5	23.8	6	22.2	1.000
Home	5	83.3	16	76.2	21	77.8	
Prenatal diagnosis							
Yes	4	66.7	11	52.4	15	55.6	0.662
No	2	33.3	10	47.6	12	44.4	
Child’s age							
Younger	3	50.0	12	57.1	15	55.6	1.000
Older	3	50.0	9	42.9	12	44.4	

Listed *p* values are based on comparisons of the groups using Fisher’s exact test; *p* < 0.05 is considered significant

Parents of children with a long hospital stay (mean 88 days compared with mean 25 days) showed a significantly higher total level of parental stress (*p* = 0.04) with significantly higher levels within the factor Parental Health (*p* = 0.03). Having a history of a prenatal diagnosis of CDH, compared with parents of children with a postnatal diagnosis of the malformation, resulted in a significantly higher experience of parental stress within the Parental Health factor (*p* = 0.01) (Tables 3, 4). There was no significant difference in parental stress between parents with younger (2–5 years) versus older (6–8 years) children at the time of filling out the questionnaire. Parents with a lower educational degree showed a significantly higher level of parental stress within the factors Social Isolation (*p* = 0.03) and Relationship with Spouse (*p* = 0.01).

## Discussion

The core findings in this study were that parents of children born with CDH who required ECMO support and/or had a long hospital stay showed a high level of parental stress. Children who required ECMO support and had a long hospital stay represented a more severely ill group of children (Table 6), which indicates a correlation between severity of the child’s malformation and the level of parental stress. Additionally, mothers and fathers scored differently, with the mothers scoring higher parental stress than the fathers. We also found an association between parental stress and receiving a prenatal diagnosis of CDH. A parent’s educational level was associated with parental stress in some of the factors.

**Table 6:** Patient characteristics of children with CDH who required/did not require ECMO treatment

	ECMO treatment *n* = 12	No ECMO *n* = 16
Prenatal diagnosis (%)	9 (75)	6 (38)*
Patch repair (%)	12 (100)	4 (25)*
LOS (days) median ± SD	62 ± 74	26 ± 19

Listed *p* values are based on comparisons of the groups using Fisher’s exact test; *p* < 0.05 is considered significant

According to a Swedish nationwide representative sample, a total score of all the five scale scores was calculated: mean 2.52 (SD 0.56). The mean for role of restriction was 3.42 (SD 0.82), while all the other scales had a mean below 3, ranging between 2.05 and 2.61 (SD range between 0.68 and 0.94) [28]. An interesting result is that the study population in general, and all the examined subgroups, reported a total lower parental stress than the Swedish nationwide representative sample, with the exception of parents of children who required ECMO support. Even though becoming a parent to a critically ill child is a severely challenging life event, there are many other regulating stressors that influence parental stress such as social support, single parenting, domestic work load, parity, care-taking issues, the mother’s age and educational level [29, 30]. Becoming a parent to a child with CDH could not be stated as a single isolated down-regulating stressor of parental stress according to the findings in this study. We did, however, find risk factors for parental stress within our study group.

More than half of the children with CDH in this study were detected prenatally, which seemed to influence negatively on parental stress within the Parental Health factor. It is commonly known that CDH is a life-threatening condition that often leads to an intensive, uncertain start in life, and the assumption that foreknowledge of a congenital malformation is beneficial for parents-to-be is questioned. Skari et al. found that a prenatal diagnosis of a congenital malformation is a single independent predictor of acute parental psychological distress after birth when compared with parents who received a postnatal diagnosis [21]. Moreover, children with a prenatal diagnosis of the malformation seem to have a more severe condition compared with infants diagnosed after birth [20]. Furthermore, Kaasen et al. showed that maternal psychological distress shortly after the detection of a fetal malformation is related to the severity of the anomaly, diagnostic and prognostic ambiguity, and gestational age [18]. Severity of the malformation, including ambiguity, has similarly been described to inflect the paternal response [31]. Aite et al. studied couples undergoing prenatal consultations due to a surgical correctable congenital malformation and found no linear correlation between the severity of a malformation and the extent of parental anxiety. However, the number of antenatal consultations could reduce the level of parental anxiety [32]. Subsequently, they studied parents’ emotional and cognitive reactions and stated that antenatal information, both written and visual, should be given several times during an ongoing pregnancy because of the intense emotional distress that parents-to-be experience at diagnosis and their ability to assimilate information affects [33]. In our clinic, parents are offered several consultations during pregnancy with a multidisciplinary team, which includes a pediatric surgeon and specialist nurse, an obstetrician, a midwife and, if needed, a psychologist. What additional information parents are impacted by is, however, impossible to know because of the magnitude of information available today. In a study of parents of children born with a congenital heart defect, parents experienced the amount of reachable information as overwhelming and asked for easily accessible and reliable information sources via the Internet [34]. Fonseca et al. stated that parent’s prior knowledge is important to assess to clarify any potentially incorrect information [35].

Over the last few decades, medical care has rapidly developed for infants born with CDH with increasing survival rates up to 80–90%, and a further late mortality after the first year of life of less than 5% [36]. Long-term sequelae are often related to the severity of the malformation, whereas children who require ECMO treatment are more affected than others [37]. Even in this study, children who required ECMO treatment represented a more severely ill group, compared with those without the need of ECMO treatment. This is shown in the higher rates of prenatal diagnosis, patch repair and the longer length of hospital stay (LOS) (Table 6). Since parental stress was high within this group of parents, there might be an association between parental stress and the severity of the child’s condition. Lewis et al. investigated post-traumatic stress disorder in parents of children supported with ECMO and found a substantial number of parents who were affected by it [38].

Even though there are several prenatal measurements for predicting postnatal outcome, there are no guarantees for the future, and a child’s health depends very much on how he or she is faring after birth. Skari et al. found that mortality and the presence of associated anomalies were consistent with psychological distress at follow-up [21]. We could not confirm any differences in parental stress over time when comparing parents with younger versus older children, which could indicate that it might not change over time. However, it would have been interesting to adjust for factors such as prenatal diagnosis, associated anomalies and ECMO treatment to be able to specify any differences in parental stress over time.

The scoring of the parental stress of fathers in this study is consistent with previous findings, where fathers reported significantly lower scores compared with mothers [26, 31]. Widarsson et al. showed in a study of 320 mothers and 315 fathers of healthy children that mothers with a low educational level, without a role model and with a poor sense of coherence had a higher level of perceived parental stress [39]. Even though fathers report lower total parental stress than mothers, Skreden et al. found that they report significantly more social isolation [26]. According to a study by Fonseca et al. mothers and fathers benefitted from different kinds of social support to reduce parental stress, but there was diffusion between both parents’ adjustments, suggesting that parents affect each other and have great impact in the partner’s level of parental stress [40]. It is well known that mothers who experience a negative childbirth subsequently have fewer children and a longer interval to their second birth [41]. Additionally, a previous history of traumatic birth indicates a significantly higher risk of developing clinically important psychological distress [42].

### Methodological considerations

The main weakness of this study is the small sample size. One justification for this is that CDH is a rare malformation, which influences the sample size. The study participants were recruited from one of the largest referral pediatric surgical hospitals in Sweden, but a national collaboration sample size could have been more beneficial. The SPSQ is only valid for parents of children up to 12 years of age. Parental stress, however, seems to remain persistent over time, so many parents of older children could have been invited to participate in this study. The Swedish Parenthood Stress Questionnaire is a reliable and valid instrument for measuring experienced parental stress, but still leaves many questions unanswered. Subsequent interviews with parents would probably have provided a deeper understanding of parental stress in this group of parents and a larger amount of fathers could have been engaged to participate.

## Conclusion

Parents of children born with CDH do not report higher levels of parental stress than Swedish parents in general. Although this study is based on a small number of participants, there seems to be a relationship between increased parental stress and the severity of the child’s malformation, since parents of children who require ECMO support and/or have a long hospital stay score higher stress levels. Furthermore, mothers seem to experience higher levels of parental stress than fathers when the child is born with CDH.
